# Four Cases of Nerve Stretching

**Published:** 1879-11

**Authors:** Herman Mynter


### FOUR CASES OF NERVE-STRETCHING.

TRANSLATED FROM DANISH, BY HERMAN MYNTER, M. D.

DR. MAAG, of Copenhagen, reports two cases of obstinate sciatica, one of them connected with strong clonic contractions in musculus gluteus maximus, both of which recovered by stretching of the sciatic nerve. The disease had lasted 1½ years in one case, three months in the other, the patients being respectively nineteen and twenty-three years of age. All other treatment had proved useless. An incision two inches long was made in the direction of the nerve, commencing at the lower margin of the musculus gluteus maximus, just between the trochanter major and the tuberositas ischii. The nerve was exposed and brought forward in the wound by aid of a hook, and traction, both in the central and peripheral direction, was made for a few seconds with considerable force. The wound did not heal in any of the cases by first intention; in one of them abscesses occurred on the femur. But the pains and contractions disappeared immediately, and both patients were discharged perfectly cured.

The two other cases are reported from the city hospital at Copenhagen, for affections of the accessory nerve. The first case was that of a woman thirty-one years of age, who six years previously, after an attack of pneumonia, suffered from a chronic spasm of the accessory nerve, which resisted all treatment. An incision was made, under antiseptic precautions, two inches long, at the sterno-cleido-mastoid muscle, lower third. The fascia colli was divided by crucial incision and the nerve exposed. The nerve was stretched, with a strabismus hook with considerable force, in central and peripheral direction, and a piece of the nerve twelve millimeters long was resected. The contractions commenced again after the cessation of the narcosis, but ceased after 1/4 hour and have not since reappeared. The wound healed by first intention. Both the sterno-cleido-mastoid and cucullaris muscles contracted normally, and neither voice nor respiration were affected by the operation. The second case was that of an unmarried woman, thirty-one years of age, who for four years had suffered from chlorosis, and for one year and a half from a chronic spasm of the accessory nerve, which increased steadily in spite of all treatment. The operation was performed in the same way, but one and a half centimeters of the nerve were resected. During the first month, following the operation, the contractions continued, although in a less degree, and then disappeared perfectly. The motions of the head are free and normal. In this case, also, the wound healed by first intention, and the cicatrix was scarcely visible.—*Nordiskt Medicinskt Archiv.*

